# Cognitive deficits and white matter abnormalities in never-treated first-episode schizophrenia

**DOI:** 10.1038/s41398-020-01049-0

**Published:** 2020-11-02

**Authors:** Mi Yang, Shan Gao, Xiangyang Zhang

**Affiliations:** 1 The Clinical Hospital of Chengdu Brain Science Institute, MOE Key Lab for NeuroInformation, University of Electronic Science and Technology of China, Chengdu, China; 2 School of life Science and technology, University of Electronic Science and Technology of China, Chengdu, China; 3 The Fourth People’s Hospital of Chengdu, Chengdu, China; 4 CAS Key Laboratory of Mental Health, Institute of Psychology, Chinese Academy of Sciences, Beijing, China; 5 Department of Psychology, University of Chinese Academy of Sciences, Beijing, China

**Keywords:** Neuroscience, Schizophrenia

## Abstract

Cognitive impairment is viewed as a core symptom of schizophrenia (SCZ), but its pathophysiological mechanism remains unclear. White matter (WM) disruption is considered to be a central abnormality that may contribute to cognitive impairment in SCZ patients. However, few studies have addressed the association between cognition and WM integrity in never-treated first-episode (NTFE) patients with SCZ. In this study, we used the MATRICS Consensus Cognitive Battery (MCCB) to evaluate cognitive function in NTFE patients (*n* = 39) and healthy controls (*n* = 30), and associated it with whole-brain fractional anisotropy (FA) values obtained via voxel-based diffusion tensor imaging. We found that FA was lower in five brain areas of SCZ patients, including the cingulate gyrus, internal capsule, corpus callosum, cerebellum, and brainstem. Compared with the healthy control group, the MCCB’s total score and 8 out of 10 subscores were significantly lower in NTFE patients (all *p* < 0.001). Moreover, in patients but not healthy controls, the performance in the Trail Making Test was negatively correlated with the FA value in the left cingulate. Our findings provide evidence that WM disconnection is involved in some cognitive impairment in the early course of SCZ.

## Introduction

Impaired cognitive function is viewed as a fundamental feature of schizophrenia (SCZ)^1–3^. Cognitive declines exist in the prodromal stage of psychosis and persist throughout the course of illness regardless of how symptoms change^4–6^. Patients show different cognitive spectrum deficits including global cognitive functioning, executive functioning, processing speed, working memory, verbal fluency, attention, and social cognition^7–9^. These impairments influence various dimensions of functional outcome including independent living ability, social functioning, and occupational functioning^10^, and have more negative impacts on brain function than the clinical symptoms of SCZ^11,12^. However, as the pathophysiological mechanisms of cognitive impairment in SCZ patients are not clear, adjunctive pharmacological treatments only produce very limited efficacy^13,14^.

Diffusion tensor imaging (DTI) is a promising approach for measuring microstructural changes with neuropathology, which can quantify the fiber orientation and the white matter (WM) integrity pathways within neural networks^15–17^. Fractional anisotropy (FA), a common DTI measure of directional dependence of water diffusion, reflects fiber structural integrity, fiber tract coherence, degree of myelination, and packing density in WM^18,19^. Widespread FA reduction has been observed in several brain regions and fiber tracts in SCZ patients relative to healthy controls^20–22^. A meta-analysis^23^ has shown WM tract abnormalities in chronic SCZ, with lower FA in the medial frontal/anterior cingulate cortex, anterior corpus callosum, left temporal WM, and right anterior limb of the internal capsule, indicating disconnections within affected gray matter regions of SCZ patients.

Previous studies about WM FA mainly used patients with chronic SCZ and there were relatively few studies using first-episode SCZ patients^24^. Yao et al.^25^. identified consistent WM FA reduction in first-episode patients in the left deep temporal lobe, including the inferior fronto-occipital fasciculus and inferior longitudinal fasciculus. Based on these neuroimaging results, the disconnection hypothesis of SCZ has been put forward, proposing that SCZ arises from functional deterioration of the brain caused by lack of connections between different regions^26–28^. However, the findings have been inconsistent. Although most previous studies indicated lower FA in first-episode patients^29–33^, other research failed to replicate the finding^34–36^ or even found elevated FA^37^.

WM fiber tracts are the neural architectures supporting high-speed interactions between different regions of the brain, and thus clinical and cognitive symptoms related to SCZ could be attributed to the alterations of WM pathways^38^. Previous studies have revealed the association between WM connections and cognitive performance in SCZ patients^39–43^. For example, executive and motor functioning deficits are linked to reduced WM integrity in the temporal and frontal connectivity in first-episode SCZ patients^32^. Worse visual and verbal learning abilities, as well as processing speed, associate with lower FA values in the left inferior longitudinal fasciculus and left inferior fronto-occipital fasciculus in SCZ^38^. These findings suggest that disconnectivity of WM tracts may contribute to cognitive deficits in SCZ and provide support for the disconnection hypothesis of SCZ. However, there have also been divergent findings, possibly due to the wide heterogeneity in the study populations and in the imaging methodologies used. In particular, previous DTI studies have mainly evaluated SCZ patients with anti-psychiatric treatment, which is an important confounder and may impact WM microstructure features and brain anatomy^44,45^.

To our best knowledge, no study has investigated the relationship between voxel-based DTI measures and cognitive performance in never-treated first-episode (NTFE) patients with SCZ. Evaluating NTFE patients has particularly advantages for understanding the neurobiology mechanism of SCZ, which can minimize the confounding effects of drug treatment, disease duration, and comorbidities caused by illness chronicity^46^. Therefore, our study involved NTFE patients in measuring FA and cognitive function to examine the relationship between WM disruption and cognitive impairment in SCZ.

## Methods

### Subjects

A total of 39 Chinese Han NTFE in-patients with SCZ were recruited from Beijing Hui-Long-Guan hospital, a largest public psychiatric hospital in Beijing with almost 1400 beds. Patients were assessed at the time of admission and during 3~6 months of follow-up to establish a DSM-IV diagnosis of SCZ. Inclusion criteria for SCZ group were as follows: (1) in an acute episode period and met the diagnostic criteria of SCZ, which was conducted by two independent and experienced psychiatrists using Structured Clinical Interview for DSM-IV; (2) the symptom duration shorter than 60 months; (3) no history of taking either antipsychotic or non-antipsychotic medications; and (4) age between 18 and 40 years old. In addition, in this study, the definition for first episode was first onset of psychotic symptoms. The mean age in patients group was 28.9 ± 10.2 years and the mean illness duration was 26.6 ± 19.3 months (Table 1).

**Table 1:** Demographics and cognition of first-episode patient and healthy control subjects.

	Schizophrenia (*n* = 39)	Control (*n* = 30)	*F*/*X*^2^	*p*
Age	28.9 ± 10.2	27.5 ± 7.9	0.39	0.54
Gender (male/female)	16/23	13/17	0.04	0.85
Education	12.4 ± 3.1	12.3 ± 4.0	0.03	0.87
MCCB				
MSCEIT	50.6 ± 10.4	52.4 ± 7.7	1.02	0.32
Category fluency	52.1 ± 10.8	58.0 ± 15.5	3.28	0.08
Symbol coding	41.2 ± 9.7	65.2 ± 9.3	96.2	<0.001
Trail Making A	46.5 ± 8.2	58.8 ± 8.4	32.1	<0.001
CPT-IP	40.9 ± 11.1	58.7 ± 8.0	58.0	<0.001
Spatial span total	41.3 ± 15.3	62.3 ± 12.4	38.8	<0.001
Digital sequence test	45.3 ± 11.9	61.9 ± 9.2	40.3	<0.001
HVLT-R total	50.6 ± 10.2	63.7 ± 8.9	34.9	<0.001
BVMT-R total	47.9 ± 9.8	60.8 ± 8.9	27.6	<0.001
Mazes (NAB) total	43.7 ± 10.0	64.5 ± 10.0	70.4	<0.001
MCCB total	44.5 ± 10.6	67.4 ± 11.8	64.9	<0.001

*BVMT-R* Brief Visuospatial Memory Test-Revised, *CPI-IP* Continuous Performance Test-Identical Pairs, *HVLT-R* Hopkins Verbal Learning Test-Revised, *MCCB* Measurement and Treatment Research to Improve Cognition in Schizophrenia (MATRICS) Consensus Cognitive Battery (MCCB), *MSCEIT* Mayer–Salovey–Caruso Emotional Intelligence Test, *NAB* Neuropsychological Assessment Battery.

Thirty healthy controls were recruited by advertising in the local community. The age, gender, and education in control group were matched with the above SCZ group (Table 1). None of subjects in control group had an individual or family history of mental disorders.

We collected all subjects’ medical history and physical examination and laboratory test results through clinical records. Exclusion criteria for all subjects were as follows: (1) with major medical illness; (2) with a history of drug or alcohol abuse/dependence; and (3) without a written informed consent. In addition, all SCZ and control subjects were right-handed.

All subjects gave written informed consent before entering this study. The research proposal was approved by the Institutional Review Committee of Beijing Hui-Long-Guan Hospital.

### Cognitive tests

Cognitive functioning was measured by the standardized neurocognitive battery called the “Measurement and Treatment Research to Improve Cognition in Schizophrenia” (MATRICS) or the “MATRICS Consensus Cognitive Battery” (MCCB)^47,48^. The MCCB includes ten standardized tests measuring functions in seven cognitive domain as follows: (1) speed of processing (Trail Making Test Part A, BACS Symbol Coding Test, Category Fluency Test); (2) attention/vigilance (Continuous Performance Test-Identical Pairs); (3) working memory (Letter-Number Span, Wechsler Memory Scale Spatial Span); (4) verbal learning (Hopkins Verbal Learning Test); (5) visual learning (Brief Visuospatial Memory Test); (6) reasoning and problem solving (Neuropsychological Assessment Battery Mazes); (7) social cognition (Mayer–Salovey–Caruso Emotional Intelligence Test (MSCEIT) Managing Emotions Branch). The MCCB provides a score for each cognitive domain as well as a composite score derived from the seven domain scores. In this study, the Chinese version of the MCCB^49^ was employed in all subjects.

### Psychopathological assessment

Two trained experienced psychiatrists evaluated psychopathological symptoms in each patient using the Positive and Negative Syndrome Scale (PANSS), with an inter-evaluator reliability being greater than 0.8 in terms of the total score.

### Imaging acquisition and analysis

DTI data were acquired by a 3.0 Tesla General Electric scanner using an eight-channel phased array coil and a single-shot echo-planar imaging sequence. Scanning parameters were as follows: repetition time, 13,525 ms; echo time, 77.3 ms; slices, 50; thickness, 2.4 mm; gap, 2.4 mm; matrix size, 128 × 128; field of view, 256 × 256 mm^2^; *b*-value, 1000 s/mm^2^; 19 gradient directions; two averages. The *b* = 0 images were scanned three times. The preprocessing was performed applying FSL (FMRIB’s Software Library) freely available at (http://www.fmrib.ox.ac.uk/). First, with the FMRIB’s Diffusion Toolbox, FA images were from the DTI data constructed by fitting a tensor model to raw images. Brain extraction was subsequently conducted using the Brain Extraction Tool^50^. Third, with the FMRIB’s Nonlinear Registration Tool, FA data of all subjects were normalized into Montreal Neurological Institute space, employing the *b*-spline representation warp field^51^. Fourth, the normalized images were resampled into 2 × 2 × 2 mm^3^, producing a standard space version of each FA image. Fifth, a mean FA image (threshold of 0.2)^38^ was created and thinned to generate a mean FA skeleton representing the centers of all tracts common to the group. The maximum FA value obtained in a direction perpendicular to each tract was subject to each skeleton voxel. Sixth, the aligned FA map of each subject was projected onto this skeleton and the resulting data were fed into voxel-wise cross-subject statistical analyses^38^. Spatial smoothing was then undertaken using a 6 mm full-width half-maximum Gaussian kernel.

### Statistical analysis

Univariate analyses of covariance were performed on the MCCB scores to compare the cognitive performance between SCZ and the control groups, with age, gender, and education as covariates. Group-level analyses were conducted on brain areas with significantly detectable WM abnormalities in SCZ vs. control groups. FA differences were compared between groups by utilizing a parametric two-sample *t*-test implemented in the Statistical Parametric Mapping 8 software (Wellcome Department of Imaging Neuroscience, London, UK), with age, gender, and education as covariates. Contrasts were carried out to detect between-group FA changes, with a significance threshold of *p* < 0.05 corrected using false discovery rate (FDR). Pearson’s correlation analyses were performed to examine the influence of demographic features on FA values in the entire sample or healthy controls or patients respectively. The correlations between FA and clinical variables were described in our previous study^33^ and therefore were not included here. Partial correlations (controlling for age, gender, and education) were calculated between FA values and MCCB scores in the two groups, respectively. Only those mean FA values obtained from the regions with observed group differences were included. Bonferroni correction was used when multiple testing was conducted. Given that 12 components of the MCCB were measured and lower FA were identified in five brain regions, a *p*-value of 0.00083 (0.05/60) was considered significant. Finally, a stepwise multiple regression analysis was conducted to examine the relationships between cognition indicated by MCCB total and index scores, psychotic symptoms, and FA values in the five brain regions.

## Results

### Cognitive performance

The total and index scores of MCCB were respectively compared between SCZ patients and controls in Table 1. Except for non-significant differences in the MSCEIT (*p* = 0.32) and marginally significant differences in category fluency (*p* = 0.08), the cognitive scores on the MCCB total and other tests were significantly lower in patients than healthy controls (all *p* < 0.001). These differences remained significant after controlling for age, gender, and education (all *p* < 0.001).

### FA values

Compared with controls, patients displayed an overall lower FA values in five brain regions as follows: the left cingulate gyrus, right internal capsule, right corpus callosum, left cerebellum, and right brainstem (all *p* < 0.05, FDR corrected; Table 2). These values did not show any significant correlations with subjects’ age, gender, and education either in the entire sample or in healthy controls or in SCZ patients (all *p* > 0.05). In the patient group, no significant correlation was found between FA values and the age of onset, course of disease, age of hospitalization, and family history of psychosis (all *p* > 0.05).

**Table 2:** Fractional anisotropy (FA) in the brain regions showing differences between schizophrenia and control subjects.

		Fractional anisotropy			
Region	Hemisphere	Schizophrenia	Control	*t*	*p*
Cingulate gyrus	Left	0.445 ± 0.028	0.463 ± 0.026	3.62	<0.05
Internal capsule	Right	0.359 ± 0.016	0.375 ± 0.013	4.13	<0.01
Corpus callosum	Right	0.478 ± 0.028	0.493 ± 0.020	4.14	<0.01
Cerebellum	Left	0.279 ± 0.012	0.287 ± 0.017	3.59	<0.05
Brainstem	Right	0.396 ± 0.012	0.409 ± 0.013	4.14	<0.01

Results were thresholded at false discovery rate (FDR)-corrected *p* < 0.05. All *p* < 0.01 after adjusting for age, gender, and education.

### Correlations between FA values and cognitive performance

**Fig. 1:**
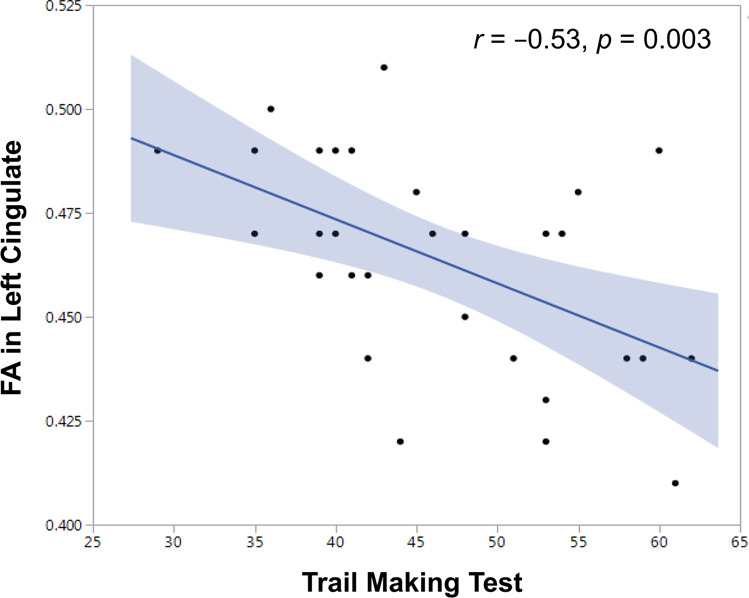
The correlation between the fractional anisotropy (FA) value in the left cingulate gyrus and performance in the Trail Making Test in never-treated first-episode patients with schizophrenia. The FA in the left cingulate was negatively associated with the Trail Making Test A index (*r* = −0.53, df = 30, *p* = 0.003).

In SCZ patients, the FA value in the left cingulate gyrus was negatively correlated with the Trail Making Test A score (*r* = −0.53, df = 30, *p* = 0.003; Fig. 1). After controlling for age, gender, education level, and duration of illness, the correlation remained significant (*r* = −0.44, df = 25, *p* = 0.023). However, it did not survive Bonferroni correction (the *p*-value should be < 0.00083). In the healthy control group, FA did not correlate with any index or the total score of the MCCB (all *p* > 0.05), probably related to a ceiling effect of MCCB scores and the small sample size.

The multiple regression analysis revealed that neither any of the MCCB indices nor its total score was contributed by FA in healthy controls (all *p* > 0.05). In the patient group, the PANSS positive symptom (*β* = −0.36, *t* = −2.17, *p* = 0.04) and FA in the left cingulate gyrus (*β* = −0.38, *t* = −2.24, *p* = 0.034) were independent contributors to the Trail Making Test index, with adjusted *R*^2^ = 0.19. However, FA did not contribute to the other MCCB indices or the total score in this group.

## Discussion

To our knowledge, this is the first study to address the association between cognitive function and WM integrity in NTFE patients with SCZ in comparison to healthy individuals. The present study had three main findings. (1) The patients showed significant disruption in WM integrity indicated by widespread reduced FA in five brain regions, involving the cingulate gyrus, internal capsule, corpus callosum, cerebellum, and brainstem, in the early stage of SCZ. (2) The patients displayed widespread neurocognitive impairment, and the total score of the MCCB and almost all of its subscales except for the MSCEIT index were lower than those in the control group. (3) FA in the left cingulate gyrus negatively correlated with the Trail Making Test index in the patient group, suggesting that WM disconnection may be one of the causes of cognitive deficits in SCZ.

Widespread FA reduction in the five brain regions in NTFE patients with SCZ is in line with most previous findings^29,31,52^, in particular those obtained in first-episode SCZ^30,32,33,53,54^, which indicates that WM abnormalities already exist at illness onset. There is accumulating evidence, including our current findings, for lower FA in multiple brain regions, suggesting that WM interruptions in SCZ are widespread rather than focal^30,55^. These findings also support the disconnection hypothesis of SCZ^56^, suggesting that the widespread disruption of WM integrity might be associated with the pathogenesis of SCZ.

Cognitive performance on the MCCB in NTFE patients with SCZ was overall worse than that in healthy controls, with significant group differences in eight of the ten scores and marginally significant group differences in category fluency. This is consistent with the majority of research on cognition in patients with chronic and first-episode SCZ^7–9,57,58^, including previous studies in Chinese patients^59^ as well as those in the United States using the MCCB^60,61^. Our findings suggest that SCZ patients have neuropsychological damage in the early stage of the disorder. The impairment in cognitive function seems to be an intrinsic characteristic of SCZ and occurs at illness onset.

Furthermore, we found that FA values in the left cingulate gyrus were negatively associated with the Trail Making Test index of the MCCB in NTFE patients with SCZ. This correlation remained significant after controlling for demographic and clinical variables. It is in line with previous evidence. The Trail Making Test examines processing speed and executive function^62^ and structural changes in the cingulate gyrus have been shown to be related to this cognitive domain^63,64^. A number of studies have reported the association of abnormal WM integration with neurocognitive deficits SCZ^39,40,42,43,65,66^. The findings involve various domains of cognitive functioning, including attention^67^, working memory^68^, processing speed^69^, language and visual learning ability^38^, executive and motor function^32^, and facial emotion perception^70^. There has also been evidence that FA measurements predict the global neurocognitive performance in SCZ patients and healthy subjects, notably for reaction time indicating a complex constellation of WM disruption responsible for altered behavioral performance in SCZ patients relative to healthy individuals^55,71^. Taken together, these findings, including ours in the present study support the proposal that the disruption of WM connectivity is related to neurocognitive deficits in SCZ.

However, it is noteworthy that WM disruption in SCZ may be observed in different brain areas due to the regional specificity of neurocognitive function in SCZ^55^. For example, lower FA in the left inferior longitudinal fasciculus and left inferior fronto-occipital fasciculus was shown to associate with the weakening of processing speed and language and visual learning^38^. Working memory performance was found to be correlated with FA changes in the cingulate fasciculi^72^ and the left superior longitudinal fasciculus^41^, as well as the covariance of cortical thickness between the posterior cingulate gyrus and ventral medial prefrontal cortex^73^. Lower FA in the cingulate gyrus was correlated with lower intelligence^74^. Facial emotion perception associated with FA in a variety of brain regions, including left forceps major, inferior longitudinal fasciculus, inferior fronto-occipital fasciculus, left splenium of the corpus callosum, and left longitudinal fasciculus, but no correlation was found in healthy individuals^70^. Interestingly, Roalf et al.^71^ reported that better general cognitive ability, indexed by lower across-task within-individual variability for performance speed on a computerized neurocognitive battery, was correlated with lower FA in the left cingulum bundle and left frontal-occipital fasciculus in healthy subjects, but not in SCZ patients. A recent study involving patients with SCZ, bipolar disorder, and major depressive disorder found that there was no significant association between reduced FA and cognitive performance in SCZ and bipolar disorder, while FA in the corpus callosum and right cingulum was significantly correlated with attention and cognitive composite performance in patients with major depressive disorder^75^. Therefore, the findings on WM-cognition association may vary greatly between studies despite the well-established hypothesis that WM tracts or certain brain structures are implicated in cognitive deficits in SCZ.

There is another point noteworthy, i.e., the negative association of the FA values in the left cingulate in SCZ patients with the performance on the Trail Making Test, which is against our prediction. Although lower FA in the cingulate is common in SCZ^76^, it has been shown to be associated with poorer ability on attention orientation^77^, with longer response time and higher response time variability in the Stroop task^71^. Based on the evidence, one would postulate a positive correlation between FA in the left cingulate and the Trail Making Test index. However, there is also evidence that less impaired cognition could be predicted by either higher or lower FA in certain brain areas in SCZ, with better neurocognitive performance related to more diffusion characteristics (negative correlation) and greater directionality (positive correlation) of regional WM^55,71^. The high heterogeneity of the association between brain connectivity and cognitive functioning in SCZ may reflect the complexity of information processing in the brain. For example, lower FA in particular areas may represent brain regions with cross-fibers^78^ or those of diffused regional connectivity with the surrounding cortex^79^, which may both be required for optimal cognitive functioning^55^. In our study, the microscopic neuroanatomical substrates of FA alterations in regional WM that might explain the negative correlation between FA values in the left cingulate and the Trial Making Test are unknown. Lower FA may reflect the changes of myelin structure, myelin content, or axon diameter^76^.

In summary, compared with health controls, NTFE patients with SCZ displayed lower FA in five brain areas, involving the cingulate gyrus, internal capsule, corpus callosum, cerebellum, and brainstem in, suggesting that the widespread disruption of WM integrity may contribute to the pathogenesis of SCZ. Also, patients showed a series of cognitive deficits at the early stage of illness. Interestingly, we found that lower FA in the left cingulate was associated with better performance in cognitive processing speed and executive functioning. However, these results need to be carefully considered for the following three reasons. (1) FA in the left cingulate was correlated with only one of the ten subscores (Trail Making Test) in the MCCB and the multiple comparisons did not hold up with Bonferroni corrections, although there were significant differences in the MCCB index and total scores between patient and controls. (2) The correlational finding is contrary to our expectation, with a negative rather than positive association between the FA values in the left cingulate and the Trail Making Test performance. We speculate that this negative association may be due to the more diffuse properties of regional WM in the left cingulate, leading to better performance in cognitive processing speed and executive functioning. (3) Although some related factors were adjusted in the main analyses, many other important factors associated with FA values, clinical symptoms and cognition were missing. These factors are especially important as a number of them are at more severe levels in patients with first-episode SCZ, such as stress, anxiety, depression, and sleep disorders, which should be remedied in future studies. (4) The voxel-based morphometry used in the data analysis in the current research has been considered as a substandard approach in DTI studies^80,81^. However, tract-based spatial statistics^82^ is considered to produce more rigorous results in our future DTI studies. (5) Future studies need to use a large sample of NTFE patients with SCZ to conduct longitudinal and prospective research on how cognition is related to brain connectivity in SCZ.
